# Do effects of common case-mix adjusters on patient experiences vary across patient groups?

**DOI:** 10.1186/s12913-017-2732-z

**Published:** 2017-11-22

**Authors:** Dolf de Boer, Lucas van der Hoek, Jany Rademakers, Diana Delnoij, Michael van den Berg

**Affiliations:** 1 Netherlands Institute for Health Services Research, Otterstraat 118-124, 3513CR Utrecht, The Netherlands; 2 TRANZO, University of Tilburg, Tilburg, The Netherlands; 3 Department of Family Medicine, CAPHRI (School for Public Health and Primary Care), Maastricht University, Maastricht, The Netherlands; 4 Department of Social Medicine, Academic Medical Centre, University of Amsterdam, Amsterdam, the Netherlands

**Keywords:** Patient experiences, Case-mix adjustment, Quality of care

## Abstract

**Background:**

Many survey studies in health care adjust for demographic characteristics such as age, gender, educational attainment and general health when performing statistical analyses. Whether the effects of these demographic characteristics are consistent between patient groups remains to be determined. This is important as the rationale for adjustment is often that demographic sub-groups differ in their so-called ‘response tendency’. This rationale may be less convincing if the effects of response tendencies vary across patient groups. The present paper examines whether the impact of these characteristics on patients’ global rating of care varies across patient groups.

**Methods:**

Secondary analyses using multi-level regression models were performed on a dataset including 32 different patient groups and 145,578 observations. For each demographic variable, the 95% expected range of case-mix coefficients across patient groups is presented. In addition, we report whether the variance of coefficients for demographic variables across patient groups is significant.

**Results:**

Overall, men, elderly, lower educated people and people in good health tend to give higher global ratings. However, these effects varied significantly across patient groups and included the possibility of no effect or an opposite effect in some patient groups.

**Conclusion:**

The response tendency attributed to demographic characteristics – such as older respondents being milder, or higher educated respondents being more critical – is not general or universal. As such, the mechanism linking demographic characteristics to survey results on patient experiences with quality of care is more complicated than a general response tendency. It is possible that the response tendency interacts with patient group, but it is also possible that other mechanisms are at play.

## Background

One of the major challenges in health care is to gather valid and reliable information on quality of care. It is commonly accepted that, at least in part, the patient perspective should be included in information on quality of care, as care should ultimately create value for patients [1]. A common way to include the patient perspective in health care quality assessment is to measure patient experiences and patient satisfaction using surveys [2–5]. Subsequently, survey results may be used as indicators of quality of care and compared between providers [4, 5].

Indicators of quality of care may be used by patients when choosing a health care provider, by commissioners when contracting providers or by providers themselves for quality improvement. For each of these purposes it is essential that the indicators are valid and reliable. Accordingly, comparisons of survey results between providers may not be confounded by factors for which providers should not be held accountable. An often raised issue in this context is that differences in scores between providers may be explained by differences in case mix [5, 6]. This occurs when there are differences between providers regarding the characteristics of their patient populations that may influence the experiences patients report and for which the provider should not be held accountable. A common example is that older patients are generally more positive about health care. Such a response tendency may lead to overestimation of quality scores for providers with older patient populations. Typically, statistical adjustments are performed for age and some other variables to address this issue when comparing patient experiences between providers [7].

Statistical adjustment for differences in case mix often include demographic variables such as age, gender, educational attainment and self-reported health [7]. The underlying rationale for using these variables is that they would reflect a response tendency where demographic subgroups of patients who have the same experiences, may still provide different responses because some patients may just be more generous/optimistic than others in providing positive responses, while others are more negative and critical [5, 7, 8]. In addition, information about these characteristics is very easy to collect as each variable generally only requires one additional question in the survey. Other variables such as comorbidity, family history of disease or health literacy generally require quite a number of additional survey items [9, 10]. Accordingly, it is virtually common practice to include demographic characteristics as potential case-mix adjusters as it is easy to do and provides some comfort in response to the criticism that differences between health care providers in the case mix of their patient populations may confound comparisons between providers.

When considering demographic characteristics as possible case-mix adjusters when comparing health care providers, the question arises whether these variables should always be included in the models, even if their coefficients are not statistically significant. If those variables do indeed reflect a response tendency, it may be expected that the coefficients are consistent across patient groups and that an occasional non-significant result is just a coincidence that may occur from time to time. The variables may be retained in the model on conceptual grounds or removed to keep the model as parsimonious as possible. However, if coefficients are inconsistent across patient groups it may be questioned whether these variables really do reflect a response tendency, and if not, whether it is still justified to adjust for these variables.

The consistency of the impact of demographic characteristics as case-mix adjusters for comparisons of patient experiences between health care providers has been studied on various occasions. For example, some inconsistencies between different types of care within hospitals have been shown for the effect of demographic characteristics on the rating of the doctor [7]. Similarly, evidence suggests that the effect of some demographic characteristics on various experiences of enrollees with a health plan vary by region [8]. Further, the impact of demographic characteristics on patient experiences appears somewhat inconsistent across hospitals [11] and general practices [12] and largely consistent across health plans [13]. Taken together, these studies provide evidence for some inconsistencies in the effect of demographic characteristics as case-mix adjusters for comparisons of patient experiences.

The present paper addresses the consistency of case-mix coefficients from a novel angle by focussing on consistency across patient groups that suffer from different conditions and/or receive care from different types of providers. The paper focuses on the global rating and on the demographic characteristics gender, age, educational attainment and self-reported health. The following research question will be addressed: *Do effects of common case-mix adjusters on patient experiences vary across patient groups?*

## Methods

### Data

The data we used were collected using Consumer Quality Index surveys (CQI; CQ-index). The CQ-index is a family of patient experience surveys that also includes methods for development and analyses [4, 14, 15]. Each survey is designed for a specific patient group or health care setting. Some survey items are only available in one particular CQI survey, other items occur in various surveys and some items are available in virtually all CQI surveys.

Table 1 provides an overview of the data used for analyses. Data were collected in various projects over the years 2007 to 2013 and included 32 different patient groups and 145,578 respondents. Data were collected predominantly by postal questionnaires only, or mixed mode data collection where respondents were given both the opportunity to respond by postal survey or online. The only exception concerns the patient group ‘Nursing home care’ for whom the survey was administered by trained interviewers. Further, representatives were approached for two patient groups that were not capable of filling out the survey (see Table 1). The global rating consisted of a single question on the quality of care on a scale from zero (very poor health care provider) to ten (excellent health care provider) and the average global rating per patient group varied from 7.1 (Spinal disc herniation) to 9.00 (Ambulance). The response rates ranged from 29% for physiotherapy to 98% for residents of nursing homes (see Table 1).

**Table 1:** Characteristics of the datasets used for analyses

						Age (%)			Self-rated health (%)		Education (%)			Gender (%)
Patient group	N units	N patients	Response	Global rating (M)	SD	<45	45 / 64	> = 65	Poor / fair	Good / excellent	Low	Medium	High	Male
Ambulance (2013)	132	1632	30%	9.00	1.13	14.58	35.78	49.63	38.79	61.21	31.99	44.85	23.16	49.08
Asthma (2008)		385	45%	7.00	1.78	36.94	39.58	23.48	40.36	59.64	38.83	42.55	18.62	40.27
Audiology care (2013)		1669	33%	8.19	1.23	37.89	34.53	27.58	13.80	86.20	17.68	48.04	34.28	36.19
Benign breast abnormality (2008)		918	64%	7.77	1.22	32.50	52.34	15.16	14.05	85.95	33.18	48.22	18.60	0.00
COPD (2009)		271	55%	7.67	1.59	6.77	43.61	49.62	60.69	39.31	56.49	33.59	9.92	49.62
Cancer care (2013)		712	50%	8.15	1.27	5.77	40.42	53.80	28.51	71.49	32.70	46.66	20.64	46.33
Cataract surgery (2010)	648	18,558	72%^a^	8.88	1.14	0.66	17.14	82.21	20.19	79.81	48.00	37.78	14.22	43.17
Cerebrovasculair accident (2011)		778	41%	8.13	1.49	4.82	32.07	63.10	46.27	53.73	47.43	38.34	14.23	49.35
Chronic Skin Disease (2013)	82	1109	29%	7.99	1.37	19.15	32.25	48.60	28.49	71.51	35.85	47.26	16.89	41.61
Chronic pain (2013)		820	40%	7.74	1.58	17.22	59.16	23.62	65.88	34.12	13.52	50.99	35.48	27.45
Diabetes (2009)	186	4242	72%^a^	8.36	1.57	3.14	36.68	60.18	38.99	61.01	61.15	33.45	5.40	45.12
Emergency department (2013)	268	4601	41%	7.82	1.63	28.62	38.17	33.21	33.45	66.55	26.04	47.91	26.04	48.71
Pharmacie (2009)	77	2845	46%	8.32	1.34	22.54	38.13	39.32	42.90	57.10	42.46	44.93	12.61	35.66
General Practice (2008)	216	5330	48%	8.15	1.28	38.27	37.91	23.82	24.24	75.76	30.84	40.66	28.50	36.98
Heart failure (2010)		312	61%	7.99	1.42	4.25	23.20	72.55	57.67	42.33	52.01	38.93	9.06	61.74
Hip- or knee surgery (2010)	293	6195	75%^a^	8.51	1.26	6.27	61.73	32.00	20.45	79.55	51.01	38.14	10.85	33.93
Home care, cleaning (2012)	434	26,594	70%^a^	8.37	1.42	2.44	12.08	85.49	74.11	25.89	59.04	34.47	6.50	21.12
Home care, nurses (2010)	237	4883	52%^a^	8.15	1.30	1.04	6.94	92.01	63.02	36.98	62.00	30.59	7.41	25.89
Hospital care (2009)	348	21,529	55%	7.97	1.45	22.14	31.77	46.09	41.45	58.55	47.99	40.90	11.11	42.55
Malignant breast abnormality (2008)		1111	68%	8.19	1.35	12.10	50.32	37.58	24.59	75.41	39.85	45.68	14.47	0.00
Maternal care (2010)	119	1826	52%	8.53	1.55	99.84	0.16	0.00	3.89	96.11	5.81	44.52	49.67	0.00
Maternity centre (2009)	229	1947	32%	7.61	1.10	98.82	1.18	0.00	3.24	96.76	5.88	54.07	40.05	7.07
Mental health ambulatory care (2010)	149	1404	–	7.60	1.65	55.77	38.32	5.91	33.26	66.74	22.58	50.14	27.28	39.67
Muscle disease hospital (2012)		331	57%	7.56	1.70	18.43	48.64	32.93	50.93	49.07	17.33	52.28	30.40	47.43
Nursing home care (2010)	83	13,173	98%^a,b^	7.81	1.19	0.25	2.92	96.83	55.21	44.79	72.24	22.90	4.86	73.57
Physiotherapy (2009)	195	1635	29%	8.41	1.07	33.76	46.06	20.18	34.07	65.93	20.61	48.62	30.76	38.09
Rehabilitation care (2010)	112	1942	45%	8.22	1.40	12.98	40.01	47.01	37.69	62.31	26.42	49.02	24.56	51.68
Repres. nursing home patients (2010)	102	9410	77%^a^	7.56	1.32	6.20	67.39	26.40	10.86	89.14	19.13	50.15	30.71	34.90
Repres. of young handicapped (2007)*	258	5283	–	7.87	1.19	65.26	33.90	0.84	–	–	15.13	56.31	28.56	20.91
Rheumatoid arthritis (2007)		352	60%	7.85	1.47	9.43	46.86	43.71	54.62	45.38	47.81	42.86	9.33	26.50
Spinal disc herniation (2009)	96	1452	47%^a^	7.13	1.81	30.92	51.58	17.49	34.23	65.77	32.78	47.87	19.35	50.90
Varicose veins (2009)	150	2329	67%	8.05	1.35	28.65	55.19	16.16	13.66	86.34	29.97	51.41	18.62	17.78

*Self-rated health was not available in this dataset

^a^The data collection was fragmented across various organizations and did not provide a response percentage. The response percentage shown is based on an earlier data collection in the same setting

^b^Data were collected through interviews in nursing homes

### Analyses

**Fig. 1:**
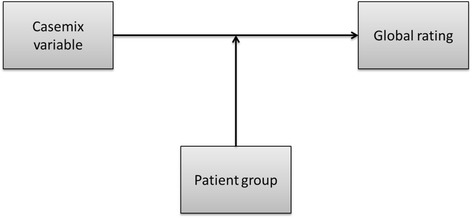
Conceptual model of the primary effects of interest. Casemix variables (gender, age, education and health) may influence the global ratings patients report for their health care provider. This influence may be differ by the contextual variable patient group

The demographic variables were all treated as categorical and dummy variables were created for each category shown in Table 1. For some of these variables, more categories were available in the data, but as the distribution of these variables is somewhat skewed in some of the patient groups (see Table 1) some of the categories were combined. An additional advantage of this strategy is that it simplifies the presentation of results. The first category of each categorical variable was used as the reference in the analyses. Linear multi-level analyses were used to assess the extent to which the impact of demographic characteristics varied across patient groups with two levels: patient group and individual. First, an empty multi-level model with random intercept was fitted to describe the dependence of observations within patient groups. Second, univariate multi-level models were fitted where each demographic characteristic was included as a fixed effect to describe the general impact of these variables across patient groups. Finally, the effect of demographic variables was allowed to vary across patient groups to assess the significance and the magnitude of this variation. This is the key analysis for answering our research question, a graphical representation of the effects of interest is provided in Fig. 1. The variation of the effects of demographic characteristics on the global rating across patient groups is illustrated by presenting the 95% expected range of coefficients, with special interest for the possibility of opposite effects across patient groups. In addition, the minimum and the maximum of the predicted coefficients for patient groups will be reported.

For 21 of the patient groups, a variable called “unit” was available in the data and referred to the health care provider of respondents (see Table 1). This allowed us to also look at a three-level model for those patient groups, with the levels patient, unit and patient group. A comparison of the results of the three-level model with the results of the two-level model for the same sample, revealed virtually identical results. Thus, although the three-level model would be better theoretically, it yielded the same results as the two-level model in reality. Since the two-level model allowed the inclusion of patient groups for which the unit variable was not available, the results of the two-level model are reported.

## Results

The empty model showed substantial and statistically significant variation of the global rating across patient groups (*p* < 0.001), which is consistent with the descriptive statistics of the global rating per patient group as shown in Table 1. The intra-class correlation coefficient was 0.092, which means that 9.2% of the variance in global rating is attributable to differences between patient groups.

Table 2 shows the results of the univariate models that included a fixed effect for covariates and a random effect for the covariates across patient groups. In each univariate model, the variation of the global rating across patient groups (constant) remained significant and the estimated 95% range of global ratings across patient groups covered more than 1.7 points in global rating. In addition, the fixed effects for the case-mix adjusters were all significant. The smallest fixed effect appeared for males compared to females (0.107) and the largest fixed effect appeared for patients older than 65 compared to patients younger than 45 (0.459).

**Table 2:** Results of univariate models for the effect of common case mix adjusters and the variance of these effects on the global rating^b^

	Coefficient^a^	SD Patient groups^a^	Estimated 95% range across patient groups	
Gender				
Constant	7.980	0.430	7.137	8.823
Female	ref	ref	ref	ref
Male	0.107	0.095	−0.079	0.293
Age				
Constant	7.746	0.458	6.848	8.644
< 45	ref	ref	ref	ref
45–65	0.228	0.159	−0.084	0.540
> 65	0.459	0.284	−0.098	1.016
Education				
Constant	8.145	0.439	7.285	9.005
Low	ref	ref	ref	ref
Medium	−0.171	0.112	−0.391	0.049
High	−0.269	0.156	−0.575	0.037
Health				
Constant	7.882	0.509	6.884	8.880
Poor/fair	ref	ref	ref	ref
Good/excellent	0.236	0.227	−0.209	0.681

^a^The coefficients and the variance of those coefficients (SD) were all significant (*p* < 0.05)

^b^N patients = 144,710–139,101; N patient groups = 31–32

The main results of interest for our research question are the standard deviations of the effect of case-mix adjusters across patient groups; the magnitude of this standard deviation is further illustrated by the estimated 95% range of case-mix coefficients across patient groups (see Table 2). For gender, the estimated 95% range of the coefficient for male varies from −0.079 to 0.293 suggesting that for most patient groups males provide higher ratings than females while for some patient groups there is no difference. In addition, it appears there may be patient groups where males provide lower ratings compared to females. For the other case-mix adjusters, a similar picture arises showing an overall effect in a negative or positive direction, while also allowing the possibility of no effect or an opposite effect in some patient groups (see Table 2).

Predicted coefficients for each patient group confirmed these observations. Indeed, for gender, the minimum of the predicted coefficients for each patient group was −.105 for patients who received mental health ambulatory care and the maximum was .234 for patients who suffered from varicose veins. For age, the minimum of the effect of age 45–65 and age > 65 was −.243 and −.143 respectively for representatives of young handicapped patients. This appeared to be an outlier as representatives of young handicapped patients was the only patient group where older respondents provider lower ratings. The maximum effects of age appeared for patients suffering from asthma where those aged 45–65 rated their care 0.476 points higher, and those aged over 65 0.998 points higher, compared to patients aged under 45. Further, the minimum predicted coefficients for education appeared for patients rating their pharmacy where medium educated patients rated their pharmacy −.443 lower, and high educated patients −.670 lower than patients with a low education. The maximum coefficients appeared for patients suffering from chronic pain, where those with a medium education provided ratings of .0520 points higher, and those with a high education .056 higher than low educated patients. Finally, the minimum predicted coefficient for self-reported health was −.333 for patients who underwent cataract surgery and the maximum coefficient was .723 for patients that suffered from spinal disc herniation.

## Discussion

This paper has demonstrated inconsistencies in the effect of case-mix coefficients on the global rating across patient groups. The effect of each coefficient on the global rating was generally positive or negative, but the variance of these effects across patient groups was also significant and showed that correction factors for the same variable may differ several tenths of the global rating between patient groups. In addition, the estimated 95% range of coefficients across patient groups generally included the possibility of no effect or an opposite effects for some patient groups. These findings indicate that the response tendency attributed to demographic characteristics [7, 8, 16] – such as older respondents being milder, or higher educated respondents being more critical – may not be general or universal. Accordingly, null findings for the effect of a common case-mix adjuster in a particular patient group may not be dismissed as occasional or coincidental, but may well reflect that in that patient group, this characteristic really does not reflect a response tendency.

It is by no means a given that demographic characteristics that are significantly associated with the variable of interest should always be adjusted for when comparing health care providers. First, it has been argued that adjustment should only take place for characteristics that are unevenly distributed across health care providers [7, 16], as comparisons between health care providers can only be confounded by variables that are unevenly distributed. Excluding variables that are evenly distributed across providers however, presents the risk of misclassifying an additional provider whose population may differ for that variable, or the risk of ignoring that variable in future analyses where the distribution of a demographic variable may have started to differ across providers. Second, the mechanism by which a demographic variable is associated with the dependent variable is of interest. As indicated, adjustment should focus on issues that may confound comparisons between providers and for which providers should not be held accountable. If differences between demographic subgroups regarding patient experiences or satisfaction are indeed a result of response tendencies it is clear that providers are not to be held accountable and that adjustment is warranted. However, it is also possible that demographic subgroups really receive a different quality of care, or have a different set of preferences [17]. On the one hand it may still be argued that adjustment is desirable when differences between demographic subgroups in the quality of care they receive is consistent across providers [18], which is often the case [19]. On the other hand, such an approach might reduce the incentive for providers to further tailor their care to meet the demands and preferences of different demographic subgroups. Accordingly, research on case-mix adjustment remains important and the focus of such research should lie beyond the issue of statistical significance of potential adjusters.

A strength of the present study is that we were able to pool the data of 145,578 respondents across 32 different patient groups where the global rating and the characteristics of respondents were all collected using virtually identical, standardized survey items. This dataset provided the opportunity to examine the consistency of case-mix coefficients across patient groups using multi-level regression, which gives a more robust and complete picture than comparing the coefficients of separate analyses for a couple of patient groups. For example, when performing analyses for each patient group separately (data not reported), many of the estimated coefficients were close to zero and not significant which raises the question if they were estimated accurately. This issue could be resolved using the current dataset and analyses by focussing on a general measure of the variance in case-mix coefficients across patient groups from the multi-level model rather than estimating these coefficients separately for each group. For the same reason, the fact that the distribution of case-mix variables was somewhat skewed in some patient groups also presented less of a problem.

The main limitation of the present study is that only the global rating could be included as a dependent variable. Accordingly, it cannot be ruled out that the effects of demographic characteristics on other dependent variables show a different level of inconsistency across patient groups. In addition, the global rating is one of the most common items in surveys of patient experiences and patient satisfaction and therefore also an important variable to address in research on case-mix adjustment.

## Conclusion

In conclusion, this paper has demonstrated a certain level of inconsistency of the effects of demographic variables in case-mix adjustment across patient groups. This finding underlines the necessity of evaluating possible case-mix adjustment for each patient group separately and suggests that other mechanisms than response tendencies may (also) explain associations between demographic characteristics and global ratings.
